# Exploring the Interplay Between Fatigue and the Oral Microbiome: A Longitudinal Approach

**DOI:** 10.3390/microorganisms13122721

**Published:** 2025-11-28

**Authors:** Laura Presutti, Madison C. Gueningsman, Blake Fredericksen, Andrew Smith, Ryan Taylor, Austin Tuckett, Christina Folsom, Rachel Wainwright, Christian Klena, Aaron C. Ericsson, Isain Zapata, Amanda E. Brooks

**Affiliations:** 1 Rocky Vista University College of Osteopathic Medicine, Englewood, CO 80112, USA; 2 Rocky Vista University College of Osteopathic Medicine, Ivins, UT 84738, USA; 3 Department of Pathobiology and Integrative Biomedical Sciences, University of Missouri College of Veterinary Medicine, Columbia, MO 65201, USA; 4 Department of Biomedical Sciences, Rocky Vista University, Englewood, CO 80112, USA; 5 Office of Research and Scholarly Activity, Rocky Vista University, Ivins, UT 84738, USA

**Keywords:** oral microbiome, microbial diversity, preclinical education, fatigue and performance

## Abstract

Fatigue is a pervasive burden for emerging medical professionals, often impacted by stress and lifestyle factors, yet insufficiently explained by these aspects alone. Considering the profound immediate and long-term consequences for physician well-being and patient care, exploring the interplay between biological factors, such as the oral microbiome and fatigue, emerges as a critical area of investigation. This exploratory longitudinal study investigates the relationship between oral microbiome diversity and fatigue in first-year medical students across four timepoints, where they provided buccal swabs and completed lifestyle and standardized stress, sleep, and fatigue assessments (PSQI, FAS, PSS). Microbiome analysis was performed using 16S rRNA sequencing and QIIME2-based bioinformatics to identify genus-level profiles and core microbiome composition. Forty-five healthy participants were assessed. Significant increases in fatigue and fluctuations in oral microbiome diversity were observed, with alpha diversity peaking mid-year before declining. Illness frequency and antibiotic use also rose, potentially influencing microbial shifts. These fluctuations may be indicative of an adaptation process where oral microbial diversity adjusts to changes in the subject’s environment, which in this case is entering medical school. Despite no clear clustering in biodiversity metrics, associations between fatigue and microbiome richness were noted, suggesting that physiologic fatigue and environmental stressors may contribute to microbial variability. Limitations of the study include a small sample size, attrition, and representativeness of the study population. This study presents a longitudinal baseline that may serve as a reference for future investigations. These findings may support the development of targeted interventions designed to modulate microbial composition as a novel approach to alleviating fatigue.

## 1. Introduction

The oral microbiome is a dynamic ecosystem encompassed by a diverse composition of microbial species influencing health and disease. There is a complex interplay between microbial dysbiosis and systemic inflammation, a fundamental driver of chronic disease [1]. Systemic inflammation is widely recognized as a key contributor to fatigue, a common symptom and often the most debilitating manifestation of chronic disease. This well-established connection could potentially link the effects of oral microbial imbalance to this pervasive symptom [2,3,4]. Although inherently subjective, fatigue is multifactorial and patient-specific, making it a particularly complex chief complaint for physicians to treat [5]. Despite many known contributors to fatigue, the underlying biological mechanisms have not been thoroughly elucidated [6,7]. The patient-specific and poorly defined nature of fatigue raises the possibility of an interconnection with the oral microbiome.

The oral cavity is the primary portal of entry for microbes to colonize and invade the human body [8]. The microbial composition within the oral cavity includes 700 types of microbes that vary amongst individuals based on maternal transmission at birth, diet, oral hygiene, medications, and other environmental factors [8,9]. These composition profiles can be compared to a reference set of healthy microbial profiles established by the Human Microbiome Project [9,10,11]. The oral microbiome plays a significant role in modulating immune responses that can trigger low-grade systemic inflammation, leading to a diffuse hyperinflammatory state. The downstream impact of oral microbial shifts has garnered profound interest over the years. Extensive research has established an association between oral microbial dysbiosis and diseases such as diabetes mellitus, cancer, Alzheimer’s disease, immune-related conditions, and chronic fatigue syndrome [8,9,12,13]. High alpha diversity characterizes a healthy oral microbiome, whereas reduced diversity has been associated with systemic diseases often characterized by fatigue, highlighting a potential link between oral microbial composition and physiologic resilience [14].

While these associations are well documented in patient populations with systemic diseases, the relationship between fatigue and oral microbial shifts in healthy individuals remains largely unexplored. In addition, there is a significant gap in the literature on the dynamic processes of the oral microbiome in response to normal life stressors. Forming a baseline understanding of the evolving symbiotic relationship between the oral microbiome and normal human physiologic and cognitive stressors is a pertinent way to expand scientific knowledge on factors that modulate health and disease. Medical students are a population of particular interest given the intensity of the academic curriculum and the demanding hours of their schedule; stress, sleep disturbances, and fatigue are known consequences [15,16]. Fatigue among this group is particularly significant, as the profession’s high burnout rates contribute to ongoing physician shortages and reduced quality of patient care [17]. Given these challenges, medical students may provide a valuable model for examining how fatigue may be linked to oral microbiome dynamics in otherwise healthy individuals.

With evidence suggesting that the balance of microbiota in the mouth reflects broader health patterns, this study aims to elucidate the interplay between fatigue and the oral microbiome in the absence of chronic systemic disease, particularly in medical students in their preclinical years. Despite the oral microbiome being far more easily accessible than the gut in a clinical context, the current literature has a much stronger focus on the latter. By investigating the interplay between the oral microbiome and fatigue over time, this study aims to understand how oral microbial changes could potentially reflect evolving health states. Insights from this study may guide future development of robust interventions aimed at mitigating chronic fatigue through topical oral formulations within the affected population.

## 2. Materials and Methods

### 2.1. Experimental Design

This exploratory study aimed to evaluate changes in oral microbial diversity and fatigue in medical students over time. Participants completed four oral microbiome sample collections over a 13-month period, with the first sample collected during medical school orientation. At each timepoint, they also completed a survey via Qualtrics that included demographic and lifestyle questions, along with the Perceived Stress Scale, Pittsburgh Sleep Quality Index, and Fatigue Assessment Scale.

### 2.2. Participants and Sample Collection

The study included first-year medical students from Rocky Vista University College of Osteopathic Medicine (Colorado and Utah campuses). All participants consented voluntarily in a written form to participate in the study and were provided with details of the intervention. Each participant was randomly assigned a research ID to maintain anonymity, and analysis was performed on a de-identified dataset. This study was approved by Rocky Vista University’s Institutional Review Board (2023-122) and conducted in accordance with the ethical principles set forth in the Declaration of Helsinki.

Participants were excluded from the study if they self-reported having any chronic diseases including, but not limited to, sleep disorders, diabetes mellitus, mental illnesses, periodontal disease, and irritable bowel syndrome. Additional exclusion criteria include taking antibiotics, pre- or probiotics, proton pump inhibitors, selective serotonin reuptake inhibitors (SSRIs), serotonin–norepinephrine reuptake inhibitors (SNRIs), tricyclic antidepressants (TCAs), and metformin.

Each participant was longitudinally followed at 4 timepoints. The first timepoint occurred in July 2023 during new-student orientation to establish a pre-medical school baseline. The second timepoint occurred in August 2023, at the end of the first curriculum block. The third timepoint occurred in December 2023, at the end of the first semester. The fourth timepoint occurred in August 2024, at the start of second year. After a 30 min fasting period, participants used sterile, cotton-tipped applicators to swab the buccal area on one side near the back molars for 30 s. Swabs were placed in labeled centrifuge tubes and stored at −20 °C. Samples were sent to the University of Missouri Metagenomic Center for DNA extraction and analysis.

### 2.3. Lifestyle Survey and Standardized Assessments

At each timepoint, participants completed a survey on demographics and lifestyle factors. The survey included questions on nutrition (omnivorous, vegetarian, or vegan), caffeine consumption (coffee, energy drinks, tea, or others), alcohol consumption (beer, wine, hard liquor, or others), smoking status, oral hygiene (how often they brush and if they use alcohol-based mouth wash), physical activity (hours a week in dedicated exercise and hours a week performing leisure physical activities), and COVID-19 status. Follow-up surveys also asked participants to report changes in their lifestyle across time points.

This survey also included standardized assessment questionnaires for the Perceived Stress Scale (PSS), Pittsburgh Sleep Quality Index (PSQI), and Fatigue Assessment Scale (FAS). These standardized tools are validated in general populations and were implemented without modifications. Grading was performed according to the established guidelines.

### 2.4. DNA Extraction, 16S rRNA Library Preparation, and Sequencing

DNA was extracted using QIAamp PowerFecal Pro DNA extraction kits (Qiagen, Venlo, The Netherlands) according to the manufacturer’s instructions, with the only difference being that samples were homogenized in bead tubes using a TissueLyser II (Qiagen) for ten min at 30/s, before proceeding according to the protocol and eluting in 100 µL of elution buffer (Qiagen). DNA yields were quantified via fluorometry (Qubit 2.0, Invitrogen, Carlsbad, CA, USA) using quant-iT BR dsDNA reagent kits (Invitrogen).

Library preparation and sequencing were performed at the University of Missouri (MU) Genomics Technology Core. Bacterial 16S rRNA amplicons were constructed via amplification of the V4 region of the 16S rRNA gene with universal primers (U515F/806R), flanked by Illumina standard adapter sequences [18,19]. Dual-indexed forward and reverse primers were used in all reactions. PCR was performed in 50 µL reactions containing 100 ng metagenomic DNA, primers (0.2 µM each), dNTPs (200 µM each), and Phusion high-fidelity DNA polymerase (1 U, Thermo Fisher, Waltham, MA, USA). Amplification parameters were 98 °C (3 min) + [98 °C (15 s) + 50 °C (30 s) + 72 °C (30 s)] × 25 cycles + 72 °C (7 min). Amplicon pools (5 µL/reaction) were combined, thoroughly mixed, and then purified by the addition of Axygen Axyprep MagPCR clean-up beads and incubated for 15 min at room temperature. Products were then washed with 80% ethanol, and the dried pellet was resuspended in 32.5 µL EB buffer (Qiagen). The final amplicon pool was evaluated using the Advanced Analytical Fragment Analyzer automated electrophoresis system, quantified using quant-iT HS dsDNA reagent kits, and diluted according to Illumina’s standard protocol for sequencing as 2 × 250 bp paired-end reads on the MiSeq instrument.

DNA sequences were assembled and annotated at the MU Bioinformatics and Analytics Core. Primers were designed to match the 5′ ends of the forward and reverse reads. Cutadapt [20] (version 2.6) was used to remove the primer from the 5′ end of the forward read. Read pairs were rejected if one read or the other did not match a 5′ primer, and an error rate of 0.1 was allowed. A minimal overlap of three bp with the 3′ end of the primer sequence was required for removal. The QIIME2 [21] DADA2 [22] plugin (version 1.10.0) was used to denoise, de-replicate, and count ASVs (amplicon sequence variants), incorporating the following parameters. (1) Forward and reverse reads were truncated to 150 bases, (2) forward and reverse reads with a number of expected errors higher than 2.0 were discarded, and (3) Chimeras were detected using the “consensus” method and removed. R version 3.5.1 and Biom version 2.1.7 were used in QIIME2. Taxonomies were assigned to final sequences using the Silva.v132 [23] database, using the classify-sklearn procedure. The genus-level core microbiome was identified using MicrobiomeAnalyst 2.0 on 31 October 2025 [24]. Hierarchical clustering was performed using the unweighted pair group method with arithmetic mean (UPGMA) using MetaboAnalyst [25].

### 2.5. Statistical Analysis

Longitudinal survey and microbiome data were compiled in a single dataset for analysis. All data summaries were evaluated descriptively using frequencies and percentages for categorical and means with their respective standard deviation for continuous variables. Within the microbiome data, a permutational multivariate analysis of variance (PERMANOVA) was performed using Past 5.0 [26]. This included an FDR threshold adjustment that is included as a default for the method. Association analysis was performed using Generalized Linear Mixed Models (GLMMs), where microbiome metrics (Richness, Simpson, and Shannon) were set as the outcome and dependent variables and evaluated independently. Demographic, lifestyle, and standardized assessment variables (PSQI, PSS, and FAS) were set as independent variables. In these models, the Repeated Measurement effect was included to address the covariance across timepoints within each participant. Normality assumptions were evaluated using graphical methods in preliminary models. Missing datapoints were coded as “missing”, which would make them not be included in the analysis. All descriptive and association modeling analyses were performed in SAS v.9.4 (SAS Institute Inc., Cary, NC, USA). Significant differences were declared at a 95% confidence level.

## 3. Results

The study included 45 first-year medical students from Rocky Vista University College of Osteopathic Medicine’s Colorado and Utah campuses. From this cohort, 27 participants (60.0%) were female and 18 (40.0%) were male. The average age of the participants was 25.2 ± 2.0 years, with the youngest participant being 22 and the oldest being 30 years of age. Average BMI was 23.65 ± 2.66 with a range of 18.66 to 30.67. In this cohort, 91.1% had previous healthcare experience, 86.7% relocated to attend medical school from a different state, and 37.8% live alone while in medical school. Within this cohort, 93.3% were omnivorous, 4.4% were vegetarian, and 2.2% were vegan. For their oral hygiene, 26.7% use mouthwash and 82.2% floss regularly. A summary of the student caffeine, alcohol, and nicotine product consumption is presented in Table 1. Participant attrition occurred throughout the study, with only 23 participants remaining at the last timepoint.

Starting at the second timepoint, participants were asked to report environmental changes along with any illness they may have experienced since the previous assessment, as shown in Table 2. Reported environmental and illness frequencies increased from the initial to the last assessment. However, for those who reported being ill, fewer reported having taken time off, but more reported adding antibiotics or new medications to their regimes.

**Figure 1:**
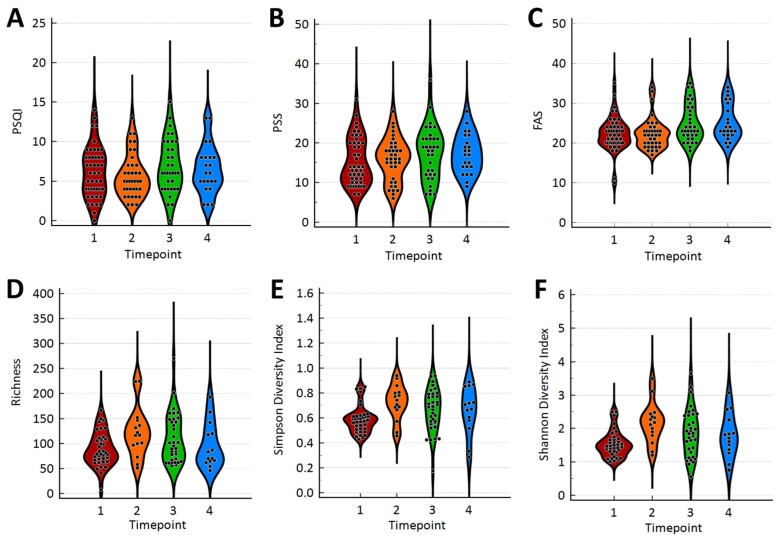
Violin plots for standardized assessment tools and microbiome biodiversity metrics. (**A**) PSQI, (**B**) PSS, (**C**) FAS, (**D**) Richness, (**E**) Simpson Diversity Index, (**F**) Shannon Diversity Index.

**Figure 2:**
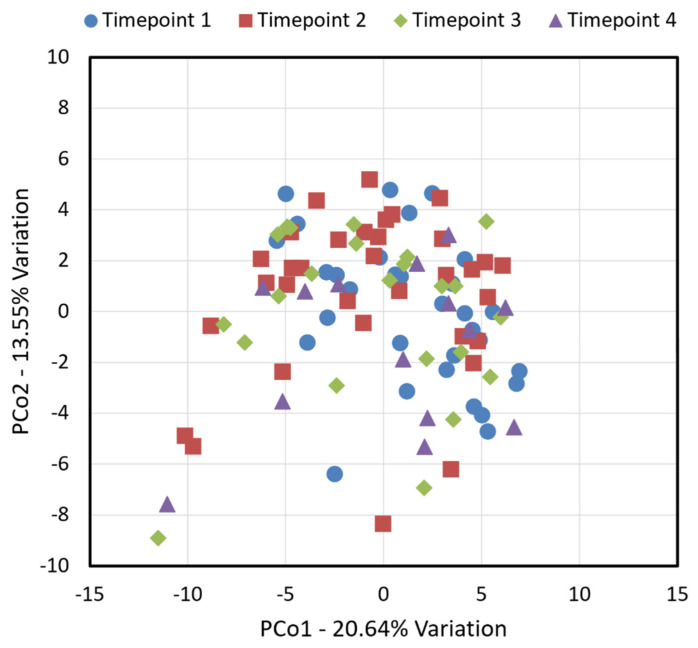
Principal coordinate analysis of microbiome biodiversity of Jaccard distances. Timepoints are coded with different colors.

Standardized tools assessments (PSQI, FAS, and PSS) are presented in Figure 1A–C. Using Repeated Measurement GLMMs to assess the effect of the timepoints, only FAS significantly increased across timepoints (*p* = 0.0072). Additionally, microbiome alpha diversity assessments (Richness, Simpson Diversity Index, and Shannon Diversity Index) are presented in Figure 1D–F. Using Repeated Measurement GLMMs to assess the effect of the timepoints, all alpha diversity assessments significantly varied across timepoints: Richness (*p* = 0.0439); Simpson Diversity Index (*p* = 0.0221); and Shannon Diversity Index (*p* = 0.0052). For all these alpha diversity metrics, an increase was first observed in timepoint 2 that then decreased, although not returning to the baseline, in subsequent timepoints. Microbiome beta-diversity using Jaccard distances is presented in Figure 2. Despite having shown differences in several of the biodiversity metrics, there is no evident cluster separation in the plots. The total variance explained with two dimensions was 34.19%.

One-way permutational multivariate analysis of variance (PERMANOVA) using Jaccard distances failed to detect an overall significant effect of time (*p* = 0.27, F = 1). However, post hoc pairwise comparisons detected a subtle difference between timepoints 1 and 2 (*p* = 0.031, F = 1.4).

**Figure 3:**
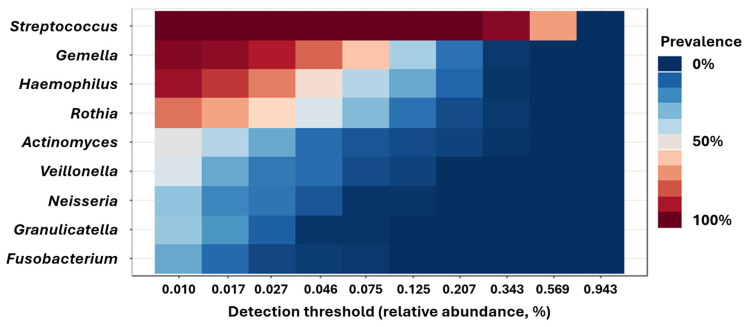
Heatmap showing the relative abundance (RA) and prevalence (scale at right) of the genus-level core microbiome, defined as genera detected in at least 0.01% mean RA and 20% prevalence.

**Figure 4:**
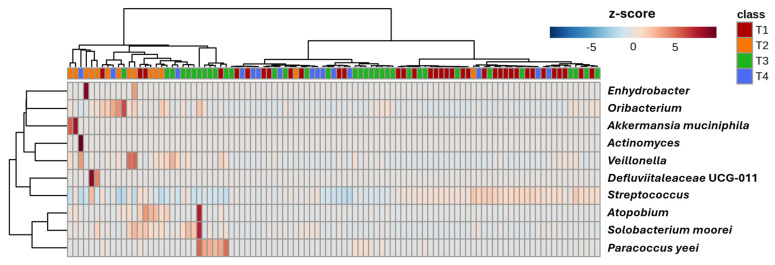
Heatmap with samples clustered using an unweighted pair group method with arithmetic mean (UPGMA) based on the 10 amplicon sequence variants yielding the lowest *p*-values in serial Kruskal–Wallis analysis of variance (ANOVA). Cells are colored according to the z-score; scale shown at right.

Taxonomic composition of samples was dominated by *Streptococcus* spp., found in 100% of the samples as the most abundant genus. The genus-level core microbiome, defined as genera detected in at least 20% of the samples (i.e., prevalence > 20%) at a minimum mean relative abundance (RA) of 0.01%, comprises only nine genera (Figure 3). *Streptococcus* was the only genus found in all samples at greater than 0.01% RA, while *Gemella*, *Haemophilus*, and *Rothia* spp. were also detected at high prevalence. To identify taxonomic features associated with the onset or progression of fatigue, we performed differential abundance (DA) testing in the form of serial Kruskal–Wallis of all 1965 amplicon sequence variants (ASVs). While several dozen ASVs yielded raw *p*-values below 0.05, none withstood correction for the false discovery rate (FDR). Nonetheless, to determine if the ASVs with the greatest DA could help discriminate sample timepoints or serve as biomarkers in the oral cavity of the onset or progression of fatigue, we performed hierarchical clustering of samples based on the RA of the 10 most differentially abundant ASVs (Figure 4). Notably, almost all samples from timepoint 2 and a subset of samples from timepoint 3 cluster independently from the other samples. This clustering is due to a relative decrease in a dominant ASV annotated to *Streptococcus* during timepoint 2 and certain timepoint 3 samples, and concomitant sporadic increases in other members of the core microbiome (*Actinomyces* and *Veillonella*) and other less abundant or prevalent taxa.

Comprehensive assessments of the microbiome and standardized tools were performed. These assessments included the demographic data reported in the baseline with and without adjustments for caffeine, alcohol, and nicotine consumption, and are presented in Table 3. In these assessments, we observed significant and consistent timepoint variability of microbiome biodiversity metrics. The only exception was in the Simpson Diversity index assessments of caffeine, alcohol, and nicotine product consumption adjusted model (*p* = 0.0694). Associations with standardized assessment tools were only detected for a single instance on FAS (*p* = 0.0488) for microbiome Richness.

## 4. Discussion

This current exploratory study investigates oral microbiome changes in healthy adult medical students experiencing fluctuations in fatigue over their first year of medical school. To ensure a comprehensive evaluation of the cohort, a baseline assessment was distributed that included standardized tools (PSQI, FAS, and PSS) as well as demographic and environmental factors such as caffeine, alcohol, and nicotine consumption. Alpha diversity, or species richness, in the cohort increased at the end of the first semester block, suggesting an adaptation period. Subsequently, there was a decrease in alpha diversity as the year progressed; however, it did not return to baseline. The FAS showed an increase in fatigue from baseline across timepoints (*p* = 0.0072). These results indicate an association between changes in the oral microbiome with increasing fatigue; however, it remains unclear how they may be interconnected and how other environmental variables play a role in these changes.

During the early stages of physician training, individuals experience a multitude of stressors. Medical students frequently experience personal distress due to environmental changes and a low sense of personal accomplishment [27]. There is a high prevalence of burnout and mental health challenges in medical students, largely attributed to their work and learning environment rather than individual factors [28]. This notion supports the cyclical changes seen in levels of depression, anxiety, and sleep quality due to fluctuations in workload throughout the academic calendar [29]. Despite individual variability, stress is also a universal response to major life transitions due to changes in family dynamics, social relationships, and place of residence [30], all additional areas of instability that add to the rigor of medical school. When compared to the general U.S. population, medical students have a higher prevalence of burnout syndrome, an entity defined by emotional exhaustion, depersonalization, and reduced personal accomplishment [27,31].

Medical trainees have certain common qualities that aid in navigating the rigor of medical training. One of those qualities, resilience, is a critical protective factor associated with less burnout and improvement in professional quality of life [32]. In comparison to the general U.S. workforce, physicians are known to have higher levels of resilience, which may help them mitigate the impact of ongoing stressors [33]. With consideration of stress in this medical school cohort, the PSS showed moderate stress at baseline and throughout the school year. Steady moderate stress throughout the school year could be due to evolving causes of stress, from the initial transitional life stressor to curriculum demands, leading to some adaptation along each phase.

In addition to psychological stressors, physical health factors such as an increase in illness frequency, with subsequent antibiotic use, were seen in this cohort and likely affected the oral microbial diversity findings. Antibiotics are known to decrease oral microbial diversity, contribute to selective overgrowth of opportunistic organisms, and aid in microbial resistance, all influencing shifts in oral microbial composition [34]. The observed shifts in microbial diversity must be interpreted in the context of these environmental changes.

A prevalent occupational hazard for the medical profession, fatigue can be subdivided based on cause, including physiologic or pathologic, categorized as acute, subacute, or chronic [6,35]. Physiologic fatigue, commonly due to extensive physical or mental activity, can be influenced by diet, physical exertion, psychological stress, and sleep. Pathological fatigue, regarded as an abnormal and persistent manifestation, is most often secondary to an underlying chronic disease [6,36,37]. For this study, we focused on physiologic fatigue, a symptom commonly experienced among medical professionals and trainees. As a result, the influence of pathologic fatigue was limited by exclusion criteria. Caffeine [38], alcohol [39], and nicotine [40] are substances frequently used as maladaptive coping strategies for stress and fatigue in this population due to the demanding nature of the profession. The relentless rigor of medical training pivots diversion behaviors toward substance use, creating a vicious cycle that perpetuates burnout and renders it inadequately treated [41]. The consequences of burnout are substantial, often contributing to physician distress, suicidal ideation, physician turnover [27], and amplified risk of medical errors [42].

The oral microbiome is an essential factor contributing to brain health. Early life adversity (ELA), defined as negative environmental exposures in the first 1000 days of life, has lifelong implications on the hypothalamus–pituitary–adrenal (HPA) axis and plays a role in shaping oral and gut microbial composition. In addition to the lasting impact of early-life psychosocial stressors on the oral microbiome, exposure to stress in adulthood can also trigger a bidirectional interaction between stress physiology and oral microbial composition, often culminating in oral dysbiosis [43]. These interactions are mediated through metabolic, immune, and neuroendocrine pathways. As a result, microbial dysbiosis can lead to neuroinflammation, affecting normal cognitive functioning [44].

Medical training is the peak time for stress and burnout among physicians. High fatigue and burnout are most common during residency and least common in earlier career stages [27]. With this known trajectory of increasing fatigue from medical school into residency, following long-term oral microbial changes through residency could yield further information to clarify a potential association between the oral microbiome and fatigue. Burnout is deemed to be cumulative over time and leads to long-term physical and psychological consequences. One of the most common causes of burnout syndrome is long-term exposure to stress [42]; therefore, it is unsurprising that it is linked to metabolic syndrome, impaired immune function, systemic inflammation, and cardiovascular disease [45]. In most clinical scenarios, fatigue is commonly attributed to an ailment, yet the severity of fatigue reported differs across diseases and does not have a direct correlation with disease progression [36,37]. This highlights the individual-specific nature of fatigue. Independent of environmental or pathological influences, individual biological factors, perhaps even the microbiome, play a powerful role, serving as a reminder that human resilience and vulnerability are uniquely shaped within each person.

### Limitations

This study has several limitations, including its small sample size, the representativeness of the study population, and the dynamic nature of the oral microbiome. The limited sample size reduces statistical power and generalizability, which may affect the reliability and applicability of the findings. In addition, the study population reflects a specific cohort with unique characteristics, further restricting generalizability. The oral microbiome itself is highly dynamic and subject to constant fluctuations, making it difficult to establish clear trends or changes in microbial composition. Moreover, numerous daily environmental factors can influence the oral microbiome and introduce potential confounding variables. Such confounders, including socioeconomic status, host genetics, recent dental procedures, and circadian rhythm variation, present obstacles when interpreting the results. Finally, systemic inflammatory status was not assessed, potentially limiting the interpretation of the observed associations.

## 5. Conclusions

This exploratory study underscores the dynamic nature of the oral microbiome and its potential association with fatigue in medical students navigating the rigors of their first academic year. Alpha diversity, or species richness, in the cohort increased at the end of the first semester block, with a subsequent decrease as the year progressed, although it did not return to baseline. An increase in fatigue was observed across all timepoints. While oral microbiome diversity and fatigue levels fluctuated in tandem, the initial rise in microbial diversity may reflect an adaptation period, especially given the steady state of moderate stress seen throughout the year. Illness frequency and the use of antibiotic and new medication increased, potentially influencing microbial shifts due to these underlying confounding factors. The findings support the need for further longitudinal research to explore microbial biomarkers of stress and resilience and their potential role in fatigue, particularly through transition into residency. Understanding these biological shifts may offer new insights into burnout prevention and wellness strategies in medical education.

## Figures and Tables

**Table 1:** Participant caffeine, alcohol, and nicotine consumption reporting.

	Timepoint 1 (Baseline) N = 45		Timepoint 2 N = 37		Timepoint 3 N = 34		Timepoint 4 N = 23	
	Freq	%	Freq	%	Freq	%	Freq	%
Consume Coffee								
No	9	20	12	32.43	9	26.47	9	39.13
Yes	36	80	25	67.57	25	73.53	14	60.87
Consume Energy Drinks								
No	28	62.22	21	56.76	16	47.06	13	56.52
Yes	17	37.78	16	43.24	18	52.94	10	43.48
Consume Tea								
No	24	53.33	24	64.86	19	55.88	11	47.83
Yes	21	46.67	13	35.14	15	44.12	12	52.17
Consume Other Caffeine								
No	36	80	33	89.19	31	91.18	22	95.65
Yes	9	20	4	10.81	3	8.82	1	4.35
Consume Beer								
No	28	62.22	24	66.67	25	73.53	17	73.91
Yes	17	37.78	12	33.33	9	26.47	6	26.09
Consume Wine								
No	34	75.56	34	91.89	30	88.24	23	100
Yes	11	24.44	3	8.11	4	11.76	0	0
Consume Hard Liquor								
No	32	71.11	34	91.89	27	79.41	21	91.3
Yes	13	28.89	3	8.11	7	20.59	2	8.7
Consume Other Alcoholic drinks								
No	42	93.33	36	97.3	34	100	23	100
Yes	3	6.67	1	2.7	0	0	0	0
Consume Nicotine products								
No	44	97.78	36	97.3	34	100	23	100
Yes	1	2.22	1	2.7	0	0	0	0

**Table 2:** Participant environmental illness and illness management reporting.

	Timepoint 2 N = 37		Timepoint 3 N = 34		Timepoint 4 N = 23	
	Freq	%	Freq	%	Freq	%
Environmental changes from previous						
No	29	78.38	31	91.18	16	69.57
Yes	8	21.62	3	8.82	7	30.43
Suffered any illness						
No	33	89.19	24	70.59	14	60.87
Yes	4	10.81	10	29.41	9	39.13
Took time off because of illness						
No	3	75	7	70	8	88.89
Yes	1	25	3	30	1	11.11
Used Antibiotics						
No	4	100	8	80	7	77.78
Yes	0	0	2	20	2	22.22
Added a new medication						
No	33	89.19	29	85.29	15	65.22
Yes	4	10.81	5	14.71	8	34.78

**Table 3:** Comprehensive assessment of microbiome diversity metrics and standardized tool assessments. These assessments are presented with and without adjustments.

		Raw			Adjusted by Demographics (Age, BMI, Gender, Previous Experience, Relocation, Living Arrangement Setting, and Diet)			Adjusted by Consumption Patterns (Caffeine, Alcohol, and Nicotine Product Consumption)		
		Estimate	Standard Error	*p*-Value	Estimate	Standard Error	*p*-Value	Estimate	Standard Error	*p*-Value
Richness	Timepoint									
	Timepoint 1	86.641	7.357	0.0340	73.416	16.190	0.0382	90.297	10.296	0.1138
	Timepoint 2	121.820	11.435		102.320	18.511		120.720	15.039	
	Timepoint 3	112.590	7.657		99.279	16.330		111.460	11.167	
	Timepoint 4	100.450	12.344		81.985	20.113		97.411	15.238	
	Standardized Assessment Tools									
	PSQI	0.597	1.624	0.7144	0.674	1.668	0.6875	1.697	1.707	0.3251
	FAS	−1.807	1.103	0.1069	−2.147	1.066	0.0488	−1.280	1.204	0.2931
	PSS	0.040	0.888	0.9644	−0.278	0.884	0.7542	0.206	0.922	0.8238
Simpson Diversity Index	Timepoint									
	Timepoint 1	0.585	0.025	0.0206	0.490	0.054	0.0087	0.570	0.035	0.0694
	Timepoint 2	0.720	0.039		0.630	0.062		0.680	0.052	
	Timepoint 3	0.668	0.026		0.574	0.055		0.655	0.038	
	Timepoint 4	0.665	0.042		0.587	0.068		0.655	0.052	
	Standardized Assessment Tools									
	PSQI	0.000	0.006	0.9689	−0.005	0.006	0.3324	0.001	0.006	0.8392
	FAS	−0.001	0.004	0.7856	−0.003	0.004	0.4802	−0.002	0.004	0.5639
	PSS	−0.003	0.003	0.2592	−0.002	0.003	0.5666	−0.003	0.003	0.3360
Shannon Diversity Index	Timepoint									
	Timepoint 1	1.516	0.099	0.0049	1.166	0.222	0.0046	1.507	0.140	0.0297
	Timepoint 2	2.152	0.154		1.774	0.254		2.045	0.205	
	Timepoint 3	1.892	0.103		1.548	0.224		1.867	0.152	
	Timepoint 4	1.838	0.166		1.509	0.276		1.807	0.208	
	Standardized Assessment Tools									
	PSQI	0.006	0.022	0.7723	−0.011	0.023	0.6212	0.017	0.023	0.4557
	FAS	−0.006	0.015	0.6724	−0.012	0.015	0.3991	−0.009	0.016	0.5712
	PSS	−0.012	0.012	0.3014	−0.006	0.012	0.6012	−0.010	0.013	0.4191

## Data Availability

The original contributions presented in the study are included in the article, further inquiries can be directed to the corresponding authors.
